# Supplementary material for: Clinical and laboratory profiles of Oropouche virus disease from the 2024 outbreak in Manaus, Brazilian Amazon
Source: PLoS Negl Trop Dis. 2025 Oct 3;19(10):e0013604. doi: 10.1371/journal.pntd.0013604 (PMC12510643; doi:10.1371/journal.pntd.0013604)
Supplement: S1 File — (DOCX) [file pntd.0013604.s001.docx]

**Supplementary Information**

**File S1*.*** Primers and probes for Oropouche virus (OROV) detection using RT-qPCR.

| **Primer/Probe** | **Sequence (5’-3’)** | **Orientation** | **Target** | **Position*** |
| --- | --- | --- | --- | --- |
| OROV_FNF | TCCGGAGGCAGCATATGTG | Sense | S segment | 98-116 |
| OROV_FNR | ACAACACCAGCATTGAGCACTT | Antisense |  | 160-139 |
| OROV_FNP | FAM-CATTTGAAGCTAGATACGG- MGB-NFQ | Sense |  | 118-136 |

Positions are indicated relative to GenBank sequence: [NC_005777.1](https://www.ncbi.nlm.nih.gov/nuccore/NC_005777.1).
