# Supplementary material for: Clinical and laboratory profiles of Oropouche virus disease from the 2024 outbreak in Manaus, Brazilian Amazon
Source: PLoS Negl Trop Dis. 2025 Oct 3;19(10):e0013604. doi: 10.1371/journal.pntd.0013604 (PMC12510643; doi:10.1371/journal.pntd.0013604)
Supplement: S2 File — (DOCX) [file pntd.0013604.s002.docx]

**Supplementary Information**

**File S2.** Primers used for Oropouche virus amplicon-based next-generation sequencing.

| **Primer** | **Sequence (5'-3')** | **Primer pool** |
| --- | --- | --- |
| OROV_L_400_1_LEFT | ACAATCTCAAAATGTCGCAGCTGT | 1 |
| OROV_L_400_1_RIGHT | CCTTCTGGATTGAACACCTCTCC | 1 |
| OROV_L_400_2_LEFT | ACTTACTGCATGACGGGAAAATGTA | 2 |
| OROV_L_400_2_RIGHT | GGATGAGTAAGCAATTCTGGGGT | 2 |
| OROV_L_400_3_LEFT | GGCAAATTTCGAGATGATGATAAATTCCA | 1 |
| OROV_L_400_3_RIGHT | TTGACATGTCACGTTCAGAGCT | 1 |
| OROV_L_400_4_LEFT | TGACAACAGGAGATTACCCCAGG | 2 |
| OROV_L_400_4_RIGHT | TGTTGCTCCCATAAGACAGTTGC | 2 |
| OROV_L_400_5_LEFT | TCATTTTGTGGAAAGTTAAAATCTCTAGCA | 1 |
| OROV_L_400_5_RIGHT | TCCCACATTTCTACACTACAGGCA | 1 |
| OROV_L_400_6_LEFT | CAAGTCACACATAACTTATCTCAAGTCAA | 2 |
| OROV_L_400_6_RIGHT | GTAGAATAGAGTGAACCAAGTTCTGCT | 2 |
| OROV_L_400_7_LEFT | TGGTTTAGTAATGCCAAGCTCAGA | 1 |
| OROV_L_400_7_RIGHT | GAGACTGCCAGCGAATTCATGA | 1 |
| OROV_L_400_8_LEFT | TCCTCTCCGGGCTTATTTCTCA | 2 |
| OROV_L_400_8_RIGHT | TCTTTTGGAATCTACTCCCTTTTGTGT | 2 |
| OROV_L_400_9_LEFT | GACAGATTTGATTAAAAAGGGCTGCT | 1 |
| OROV_L_400_9_RIGHT | TTTGCATTTTCTACTCGACTTCTCAC | 1 |
| OROV_L_400_10_LEFT | TGCCAAGCAGACTGTCAACTTAA | 2 |
| OROV_L_400_10_RIGHT | AGCTTGACTGTTTCTTTATCACTTATTTCA | 2 |
| OROV_L_400_11_LEFT | AACAATCCATCCCAGATTACACAGA | 1 |
| OROV_L_400_11_RIGHT | CCCAATCTAAAGACCCGGATTGC | 1 |
| OROV_L_400_12_LEFT | AGTGAACCAGGTGATTCTAAGTTAAGAGTA | 2 |
| OROV_L_400_12_RIGHT | GCCAGTTGCGTTTTATTTCTACTGT | 2 |
| OROV_L_400_13_LEFT | TGCTTACTACTATATTGGACCAGAGGG | 1 |
| OROV_L_400_13_RIGHT | ACAAACTCCTTGATGAAATTGGTTAGGT | 1 |
| OROV_L_400_14_LEFT | GCCAGACGACAATATAATTGAATTTTGC | 2 |
| OROV_L_400_14_RIGHT | TTCCGCACATTTCCACTGGTAT | 2 |
| OROV_L_400_15_LEFT | ACTGGATAACCCACACCACGTAT | 1 |
| OROV_L_400_15_RIGHT | CGAGGTGTTAAAAGTGATCGGGA | 1 |
| OROV_L_400_16_LEFT | ACATTCTGAGGCTTAAAATGCTTAGGT | 2 |
| OROV_L_400_16_RIGHT | GCAATCCGAAGATCTTATCATGTATGCT | 2 |
| OROV_L_400_17_LEFT | AGAATCTTTATCAATACAAAATCCAGCACA | 1 |
| OROV_L_400_17_RIGHT | AGGCTCTCAAAACAACTGCTGG | 1 |
| OROV_L_400_18_LEFT | GGACATAGCCAAGAAAGAATAGGACA | 2 |
| OROV_L_400_18_RIGHT | GTCAGTGCGCCACAGAATTCTA | 2 |
| OROV_L_400_19_LEFT | GCATCTAGGAAGGGATTTCAAGTTCG | 1 |
| OROV_L_400_19_RIGHT | ACAGGTATTCCCTTGTATGTGAACTC | 1 |
| OROV_L_400_20_LEFT | TGAAGCACTGAGACTAGTAGCACA | 2 |
| OROV_L_400_20_RIGHT | ACATCGCCTGACCGTGTCTATAA | 2 |
| OROV_L_400_21_LEFT | GTGGTTATTCTAGGCAGTTGCGA | 1 |
| OROV_L_400_21_RIGHT | TGGAAAGTATAGGTCGTCCTTGTTG | 1 |
| OROV_L_400_22_LEFT | GACCACAATTCTAGAGTCGAATCATCT | 2 |
| OROV_L_400_22_RIGHT | TGGTCATTCTCACTGCCATTGC | 2 |
| OROV_L_400_23_LEFT | AGTTAATGAAATCCACATCCTTATTGAGCT | 1 |
| OROV_L_400_23_RIGHT | AGTTCTGTGGACCACTGATTCGT | 1 |
| OROV_L_400_24_LEFT | ACTTTCTTGACATGGGGTTTTGCT | 2 |
| OROV_L_400_24_RIGHT | ACTCTGCCAGTGATCTTTTCTCG | 2 |
| OROV_S_400_1_LEFT | TCCAATAATGTCAGAGTTCATTTTCAATGA | 1 |
| OROV_S_400_1_RIGHT | GCCCTAAACTCAGCTTGCTTAATTGG | 1 |
| OROV_S_400_2_LEFT | CAGTTCCAGTCGAATCCAGTGC | 2 |
| OROV_S_400_2_RIGHT | TCTCTTGCTGCTGGGGAGAATC | 2 |
| OROV_S_400_3_LEFT | CGGCATGGATGTCAACTTTATGAAG | 1 |
| OROV_S_400_3_RIGHT | TGACATAGCTTTCCCCAACCAC | 1 |
| OROV_M_400_1_LEFT | GCAACAAACAGTGACAATGGCG | 1 |
| OROV_M_400_1_RIGHT | GCTGCTCTACATGTGTACATTTTAGG | 1 |
| OROV_M_400_2_LEFT | TCTGAGTGCAATCCAGTGCTAGA | 2 |
| OROV_M_400_2_RIGHT | ACATTGCTTCAATCATTGAGTATGGC | 2 |
| OROV_M_400_3_LEFT | TGTGGTCAGAAATCAATAAAATTCCATGC | 1 |
| OROV_M_400_3_RIGHT | GCACCTTAAGAGCTTCTGTGCA | 1 |
| OROV_M_400_4_LEFT | CCTGCCCAAATTGTCTACTTGCA | 2 |
| OROV_M_400_4_RIGHT | AAATGCTGAAAAGCCGGTTGAAT | 2 |
| OROV_M_400_5_LEFT | AGAAATGGAAGTCTTGAAGCAGTCA | 1 |
| OROV_M_400_5_RIGHT | CAAGCAATCTTCATCTGCATAAACTGT | 1 |
| OROV_M_400_6_LEFT | TGCAAATTTAGTCCAAGAGTAAACCACT | 2 |
| OROV_M_400_6_RIGHT | GCGCCAGGCAACATTATAAGGT | 2 |
| OROV_M_400_7_LEFT | AGTTTGCCCAAGATTCACATAGAATGA | 1 |
| OROV_M_400_7_RIGHT | GCCTTAAGAGCATCAGAGTCATCT | 1 |
| OROV_M_400_8_LEFT | GCTGACCTGAACACCATAATGGA | 2 |
| OROV_M_400_8_RIGHT | AAAGCAGGTGGTTTGTATGCGG | 2 |
| OROV_M_400_9_LEFT | ACGAAATTCCCAAATCTAATCCTTTTACTG | 1 |
| OROV_M_400_9_RIGHT | CCACAGAATACGATCGGCCATG | 1 |
| OROV_M_400_10_LEFT | CGCAACCAGCAGATATGCAGAC | 2 |
| OROV_M_400_10_RIGHT | GTGGAAGGTTCTTTGTGGGCTT | 2 |
| OROV_M_400_11_LEFT | AGACCCACAAAGAATTTAGCACTATGA | 1 |
| OROV_M_400_11_RIGHT | CAGGAATCTTTTCGGGGCAGTG | 1 |
| OROV_M_400_12_LEFT | AGCACAGTACCAGAAAGCATACAC | 2 |
| OROV_M_400_12_RIGHT | AGGGTCGGAAGTTGGTTAGTCT | 2 |
| OROV_M_400_13_LEFT | ACTTTGCCTCATGAAACTTATTGCAA | 1 |
| OROV_M_400_13_RIGHT | TTTGGCCCATGTTTGATCCTGT | 1 |
| OROV_M_400_14_LEFT | TGACAATTATCAGTCATGCACTAGGTT | 2 |
| OROV_M_400_14_RIGHT | ATGTCTCTCCCACAGATATGTGCT | 2 |
| OROV_M_400_15_LEFT | CTGCCATTGTAAAACAAACTGTGAAGA | 1 |
| OROV_M_400_15_RIGHT | CCGACCTATGGGCAACAGGATAT | 1 |
| OROV_M_400_16_LEFT | ACAATCAGAAGATAGATTTGTCGCAGTT | 2 |
| OROV_M_400_16_RIGHT | TGTGCTACCAACAACAATTTTTGACT | 2 |
