# Supplementary material for: Clinical and laboratory profiles of Oropouche virus disease from the 2024 outbreak in Manaus, Brazilian Amazon
Source: PLoS Negl Trop Dis. 2025 Oct 3;19(10):e0013604. doi: 10.1371/journal.pntd.0013604 (PMC12510643; doi:10.1371/journal.pntd.0013604)
Supplement: S3 File — (DOCX) [file pntd.0013604.s003.docx]

**Supplementary Information**

**File S3.** Oropouche virus sequencing data information.

| **Sample ID** | **L segment** | | | **S segment** | | | **M segment** | | |
| --- | --- | --- | --- | --- | --- | --- | --- | --- | --- |
|  | **Coverage (NT)*** | **Coverage (CDS)*** | **Average depth** | **Coverage (NT)*** | **Coverage (CDS)*** | **Average depth** | **Coverage (NT)*** | **Coverage (CDS)*** | **Average depth** |
| **05** | 98.0% | 99.2% | 2696.01 | 95.1% | 100% | 2013 | 99.3% | 100% | 3458.98 |
| **17** | 97.7% | 98.9% | 2842.35 | 95.1% | 100% | 2190.02 | 99.3% | 100% | 3221.06 |
| **24** | 94.7% | 94.7% | 2767.87 | 95.1% | 100% | 1794.95 | 99.0% | 99.7% | 3135.68 |
| **43** | 98.0% | 99.2% | 3067.4 | 95.1% | 100% | 2313.4 | 99.3% | 100% | 3748.05 |
| **509** | 94.6% | 95.7% | 2312.34 | 95.1% | 100% | 1919.3 | 99.3% | 100% | 3041.26 |
| **514** | 97.6% | 98.8% | 2532.26 | 95.1% | 100% | 1990.46 | 99.3% | 100% | 2775.44 |
| **515** | 94.7% | 95.9% | 3015.59 | 95.1% | 100% | 2165.34 | 99.3% | 100% | 3372.52 |
| **518** | 97.7% | 98.9% | 2409.3 | 94.4% | 100% | 1742.33 | 99.2% | 100% | 2850.52 |
| **559** | 98.0% | 99.2% | 2790.6 | 95.1% | 100% | 2077.94 | 99.3% | 100% | 3119.1 |
| **560** | 97.8% | 98.9% | 1982.48 | 95.1% | 100% | 1478.89 | 99.3% | 100% | 2111.38 |
| **576** | 97.7% | 98.9% | 2909.53 | 95.1% | 100% | 2332.99 | 99.3% | 100% | 3390.15 |

*Coverage against to the OROV reference sequences: L segment, M segment, S segment.
