# Supplementary material for: Clinical and laboratory profiles of Oropouche virus disease from the 2024 outbreak in Manaus, Brazilian Amazon
Source: PLoS Negl Trop Dis. 2025 Oct 3;19(10):e0013604. doi: 10.1371/journal.pntd.0013604 (PMC12510643; doi:10.1371/journal.pntd.0013604)
Supplement: S4 File — The Oropouche virus sequencing data generated during the current study are available in the GISAID repository (GISAID.org/EpiArbo). To access and view the sequences, one needs to register to the site (https://gisaid.org/register/), then LOGIN to the GISAID repository. Once you have a login, you’ll enter this area Epiarbo>Oropouche, and then click on search. You will then type in the sequence ID, of which it will appear. When you click on it (the ID), you can see the file information and download the sequences. In the case of OROV, it is an access number for the 3 segments. (DOCX) [file pntd.0013604.s004.docx]

**Supplementary Information**

**File S4.** Oropouche virus genome sequences generated in this **study and their respective accession numbers.**

Instructions:

To view the sequences, you need to register to the site (https://gisaid.org/register/) then LOGIN to the GISAID repository.

Once you have a login, you'll enter this area **Epiarbo>Oropouche**, and then click on search.

You will then type in the sequence ID, of which it will appear. When you click on it (the ID), you can see the file information and download the sequences.

In the case of OROV, it is an access number for the 3 segments.

| **Sample ID** | **GISAID arbo Accession IDs** | **Date of collection** | **Municipality** | **State** | **Country** | **Sex** | **Age** |
| --- | --- | --- | --- | --- | --- | --- | --- |
| **05** | **>hOROV/Brazil/AM-FAMERP-005/2024\|EPI_ISL_19793862\|2024-01-31** | **31/01/2024** | **Manaus** | **Amazonas** | **Brazil** | **Female** | **14** |
| **17** | **>hOROV/Brazil/AM-FAMERP-017/2024\|EPI_ISL_19793863\|2024-02-01** | **01/02/2024** | **Manaus** | **Amazonas** | **Brazil** | **Male** | **47** |
| **24** | **>hOROV/Brazil/AM-FAMERP-024/2024\|EPI_ISL_19793864\|2024-02-05** | **05/02/2024** | **Manaus** | **Amazonas** | **Brazil** | **Male** | **67** |
| **43** | **>hOROV/Brazil/AM-FAMERP-043/2024\|EPI_ISL_19793865\|2024-02-08** | **08/02/2024** | **Manaus** | **Amazonas** | **Brazil** | **Male** | **27** |
| **509** | **>hOROV/Brazil/AM-FAMERP-509/2024\|EPI_ISL_19793866\|2024-02-01** | **01/02/2024** | **Manaus** | **Amazonas** | **Brazil** | **Female** | **27** |
| **514** | **>hOROV/Brazil/AM-FAMERP-514/2024\|EPI_ISL_19793867\|2024-02-02** | **02/02/2024** | **Manaus** | **Amazonas** | **Brazil** | **Female** | **50** |
| **515** | **>hOROV/Brazil/AM-FAMERP-515/2024\|EPI_ISL_19793868\|2024-02-02** | **02/02/2024** | **Manaus** | **Amazonas** | **Brazil** | **Male** | **56** |
| **518** | **>hOROV/Brazil/AM-FAMERP-518/2024\|EPI_ISL_19793869\|2024-02-02** | **02/02/2024** | **Manaus** | **Amazonas** | **Brazil** | **Female** | **44** |
| **559** | **>hOROV/Brazil/AM-FAMERP-559/2024\|EPI_ISL_19793870\|2024-02-07** | **07/02/2024** | **Manaus** | **Amazonas** | **Brazil** | **Female** | **23** |
| **560** | **>hOROV/Brazil/AM-FAMERP-560/2024\|EPI_ISL_19793871\|2024-02-07** | **07/02/2024** | **Manaus** | **Amazonas** | **Brazil** | **Female** | **38** |
| **576** | **>hOROV/Brazil/AM-FAMERP-576/2024\|EPI_ISL_19793872\|2024-02-16** | **16/02/2024** | **Manaus** | **Amazonas** | **Brazil** | **Female** | **29** |
