# Supplementary material for: Clinical and laboratory profiles of Oropouche virus disease from the 2024 outbreak in Manaus, Brazilian Amazon
Source: PLoS Negl Trop Dis. 2025 Oct 3;19(10):e0013604. doi: 10.1371/journal.pntd.0013604 (PMC12510643; doi:10.1371/journal.pntd.0013604)
Supplement: Attachment — Submitted filename: PNTD-D-25-00611.docx [file pntd.0013604.s005.docx]

The recent surge in incidence and geographic spread of OROV infections poses an escalating threat to global public health. The authors provided high-quality data on the clinical and laboratory evaluation of OROV patients in a 2024 outbreak in Manaus, Amazonas state, Brazil. However, there are some important issues here that require further clarification by the authors.

Major

Q1: The background logic of the article is confusing, and the fourth paragraph should be put after the third paragraph.

Q2: The quality of most of the images included in the text is too blurred to see clearly, and the small images in each large image should be marked uniformly.

Q3: Figure 2 shows the frequency of symptoms and signs in OROV and dengue virus patients. But I don't see a description of Figure 2 in the results section.

Q4: The English writing is confusing and the author needs to edit it again

Minor

Q1: Page 3, line 128. Please replace “5 years” with “5yrs”.

Q2: The abbreviations of virus names in Figure 1 should be the same as in the text.
