# Supplementary material for: Clinical and laboratory profiles of Oropouche virus disease from the 2024 outbreak in Manaus, Brazilian Amazon
Source: PLoS Negl Trop Dis. 2025 Oct 3;19(10):e0013604. doi: 10.1371/journal.pntd.0013604 (PMC12510643; doi:10.1371/journal.pntd.0013604)
Supplement: Attachment — Submitted filename: Rebuttal letter.docx [file pntd.0013604.s007.docx]

Rebuttal Letter

11/08/2025.

PLOS Neglected Tropical Diseases

Dear Managing Editor,

We appreciate the opportunity to revise our manuscript PNTD-D-25-00611: *“Clinical and Laboratory Profiles of Oropouche Virus Disease and Dengue from the 2024 Outbreak in Manaus, Brazilian Amazon”*. We thank the reviewers for their insightful and constructive feedback, which contributed to enhancing the clarity, structure, and overall quality of our work.

We have carefully addressed all the comments and suggestions provided, and we believe the revisions have significantly strengthened the manuscript. We are hopeful that the revised version now meets the standards of the journal.

Below, we provide a detailed point-by-point response to the comments and questions raised by the reviewers and editors.

Once again, we sincerely thank the reviewers for their thoughtful input and the editorial team for considering our work for publication.

Sincerely,
Wuelton Monteiro, PhD
On behalf of all co-authors

AUTHOR RESPONSE

Journal Requirements:

1)	Please, provide an Author Summary. This should appear in your manuscript between the Abstract (if applicable) and the Introduction, and should be 150-200 words long. The aim should be to make your findings accessible to a wide audience that includes both scientists and non-scientists. Sample summaries can be found on our website under Submission Guidelines:
<https://journals.plos.org/plosntds/s/submission-guidelines#loc-parts-of-a-submission>

We are grateful for this correction. The Author Summary has now been added to conform to journal requirements (See Lines 58-71).

2)	We noticed that you used the phrase 'data not shown' in the manuscript. We do not allow these references, as the PLOS data access policy requires that all data be either published with the manuscript or made available in a publicly accessible database. Please amend the supplementary material to include the referenced data or remove the references.

Thanks for this observation. The phrase ‘data not shown’ has been removed.

3)	Please, upload all main figures as separate Figure files in .tif or .eps format. For more information about how to convert and format your figure files please see our guidelines: <https://journals.plos.org/plosntds/s/figures>

We have done that. They are in .tiff format and 300dpi.

4)	Tables should not be uploaded as individual files. Please remove these files and include the Tables in your manuscript file as editable, cell-based objects.

Tables have now been included inside the manuscript, EXCEPT for the supplementary tables listed as Supplementary information.

5)	We have noticed that you have uploaded Supporting Information files, but you have not included a list of legends. Please add a full list of legends for your Supporting Information files after the references list.

The list has been added in the manuscript after the references list (See lines 758-781). Please note that we have also added a new Supplementary Table 5 and Supplementary Figure 1 to the list.

6)	In the online submission form, you indicated, "The analyzed data sets generated during the study are available from the corresponding author on reasonable request." All PLOS journals now require all data underlying the findings described in their manuscript to be freely available to other researchers, either
1. In a public repository
2. Within the manuscript itself
3. Uploaded as supplementary information.
This policy applies to all data except where public deposition would breach compliance with the protocol approved by your research ethics board. If your data cannot be made publicly available for ethical or legal reasons (e.g., public availability would compromise patient privacy), please explain your reasons by return email and your exemption request will be escalated to the editor for approval.

All data and materials supporting the findings of this manuscript are presented in the paper and/or the supplementary information files. The Oropuche virus sequencing data generated and/or analyzed during the current study are available in the GISAID repository (GISAID.org/EpiArbo), using the accession numbers available in the Supplementary File_Table 4.

7)	Please, amend your detailed Financial Disclosure statement. This is published with the article. It must therefore be completed in full sentences and contain the exact wording you wish to be published.

Funding details is now amended, and included in the article. (See Funding section).

8)	Please, ensure that the funders and grant numbers match between the Financial Disclosure field and the Funding Information tab in your submission form. Note that the funders must be provided in the same order in both places as well. Currently, "University of Texas Medical Branch" is missing from the Funding Information tab.

We have excluded the funding segment associated with UTMB (See Funding section).

Addressing the Comments by Reviewers:

Reviewer #2:

- even revision on puncture, verbs and sentences construction should be performed.
- some passages are redundant and confounding, needing revision.

Reviewer #3:

- The paper is so long and confusing and relatively disorganized that it is hard to draw any conclusions from the data.
- There are multiple questions that could be addressed perhaps in more than one manuscripts on 1) the pathophysiology of disease (e.g., pathogenesis of headache and other symptoms as it relates to evolution of cytokine responses and neurological biomarkers, CNS neuroinvasion vs. inflammatory-mediated or vascular changes), 2) differences in clinical and biomarker profiles between Oropouche virus disease and dengue - e.g., are there clinical profiles that are predictive of one or the other or of severe outcomes? 3) are there genetic mutations associated with clinical syndromes or severity of disease?
- Overall, the authors need to clarify their hypotheses and organize the methods, results, and discussion accordingly.
- The paper needs to be streamlined and shortened considerably.
- The abstract doesn't make it clear which symptoms, cytokines, and biomarkers are different between Oropouche virus disease and dengue virus.

The abstract has been revised to clearly differentiate the findings between the two infections and the paper has been shortened as advised.

Methods

Reviewer #1 (assumed)

The recent surge in incidence and geographic spread of OROV infections poses an escalating threat to global public health. The authors provided high-quality data on the clinical and laboratory evaluation of OROV patients in a 2024 outbreak in Manaus, Amazonas state, Brazil. However, there are some important issues here that require further clarification by the authors.

Major

1. The background logic of the article is confusing, and the fourth paragraph should be put after the third paragraph.

We have revised and reorganized the background.

2. The quality of most of the images included in the text is too blurred to see clearly, and the small images in each large image should be marked uniformly.

This has been revised.

3. Figure 2 shows the frequency of symptoms and signs in OROV and dengue virus patients. But I don't see a description of Figure 2 in the results section.

We have included an in-text description for Figure 2 (254 - 261).

4. The English writing is confusing and the author needs to edit it again

We have revised the language in the manuscript for clarity.

Minor

1. Page 3, line 128. Please replace “5 years” with “5yrs”.

Corrected, (See Line 114).

2. The abbreviations of virus names in Figure 1 should be the same as in the text.

This had been corrected.

Reviewer #2

Study design

1. Line 128 – Why the study excluded the children under 5 years? Clinical differences may exist according to age that should be considered, evaluated and reported to the community. If ethical or other concerns exist, please clarify.

The symptoms of arboviruses (fever, headache, myalgia) are often nonspecific in this age group and can be confused with other common childhood viral infections in childhood. Furthermore, young children may not adequately verbalize neurological symptoms (such as photophobia), thereby affecting the accuracy of the data. We also point out that the serial collection of samples (D1 and D28) required multiple venipunctures, which poses ethical and compliance challenges in pediatric patients. Finally, the study aimed to better understand the difference between the two pathologies and compare variations between age groups. Future studies can better explore this variable. We can highlight these points in limitations, if needed.

2. Line 133-139 – Please make the symptoms list more concise, referring to table or results.

We have revised as suggested (see Lines 120-123).

3. Line 146 – Why molecular analysis was limited to OROV and DENV, considering the previously mentioned arboviruses (i.e.: CJKV. ZIKV…)? They are also mentioned in line 156 in molecular assays section.

We appreciate the insightful observation.

The decision to focus the molecular analysis on Oropouche virus (OROV) and Dengue virus (DENV) is grounded in the fact that these two arboviruses were responsible for the highest epidemiological burden during the 2024 outbreak in Manaus. Consequently, the primary objective of the study was to compare directly OROV and DENV.
Screening for other infections was done to identify and exclude cases with unrelated pathogens, thereby refining the final **To Analyze Group** of samples to be used in our analysis. We have endeavored to clarify the logic (See lines 149-156).

Laboratory diagnosis of OROV and dengue

4. Line 150 – Is the OROV RT-PCR a real quantitative assay, thus implying the use of calibration curve and standards?

Here we used real time PCR for detection of the virus nucleic material. The Ct (cycle threshold) values produced from known samples was used to estimate possible viral load. Positive and negative controls were also included in the assay as references. No calibration curve or standards were applied in our assays.

5. Line 151 – PRNT is a test for the detection of antibodies, not for determination of viral presence or titration. How authors used it? No result is reported in the work.

This test was used to improve on diagnostic sensitivity for OROV cases. It helped capture cases of individuals exposed to the virus at any some point before they visited the hospital on D1 to seek care for their symptoms. Together with the PCR, it was employed to capture cases that might have been missed because of absence or low viremia.

We have included in the revised manuscript a better description of the PRNT assay (See Lines 133-147), and the distribution of cases as detected by PRNT (on D28) and PCR (on D1) (See Lines 287-313; Tables 4 and 5).

6. Line 155-158 – How DENV serotype was determined?

The DENV serotyping was done through the use of primers, probes and fluorophores specific for each serotype. These primers and probes were part of DENV kits provided by the Brazilian Ministry of Health, the ZDC molecular assay kit (Bio-Manguinhos, Brazil) and Biomol ZDC kit (Instituto de Biologia Molecular do Paraná, Brazil).

We have now included this information in the manuscript (see line 151-154).

7. Why samples (or patients) positive for CHKV and ZIKV were excluded?

Again as earlier explained, the aim of the study was to understand the differences between dengue and oropouche, which were the arboviruses with the highest number of cases in the outbreak in question. Thus CHIKV and ZIKV were not in the scope of the current study.

Virus genotyping

8. Line 164-171 – Was the S RT-PCR different from the one used for OROV infection diagnosis? Why was a second RT-PCR performed, if a data was already available from first one? In addition, to be uniform the molecular analyzer should be reported also in the previous section.

They were different.

At the hospital we used real-time PCR (RT-PCR) for diagnosis, while a reverse transcription-quantitative PCR (RT-qPCR) was applied for the genotyping, at a different facility. The RT-qPCR analysis was performed using archived aliquots of the patients’ D1 samples, now confirmed to be OROV positive. The RT-qPCR analysis generated cDNA that was then used to genotype and sequence the detected virus.

We have revised the segment, and renamed the Virus genotyping section for better clarity. (See lines159-174).

9. Line 169 – The Ct value is not a mean of quantification, if anything a semi-quantification one. Quantification always need a standard curve to be performed, as mentioned before.

As we had mentioned previously, we did not perform any standard curves for viral quantification. The Ct value was used a guide to identify which samples were positive and meeting the threshold for use in cDNA synthesis.

10. Line 173-174 – Please rewrite the sentence: it is not very clear how the protocol and kit were adapted for OROV sequencing.

We have revised to “The cDNA synthesis, OROV genome amplification, and library preparation were performed using a modified version of the Illumina COVIDSeq Test (Illumina, USA), in which the original primers were replaced with a serotype-specific primer panel for OROV, designed by the Brazil-UK Centre….” (See lines 170-173).

Neurological markers and Cytokines and chemokines

11. The methods used for neurological and inflammatory markers are the same: please unify the paragraphs or synthesize them, including a reason for selecting the reported markers.

Thanks for this observation. We have revised the segment and opted to exclude the neurological assays. (See lines 206-213).

Reviewer #3:

12. Network of symptoms analysis requires more explanation. What was the question or hypothesis being tested?

Since arboviral infections tend to have overlapping symptoms, the network of symptoms analysis employed aimed to address the exploratory question “Do OROV and DENV infections differ in patterns of symptom co-occurrence and, how?”

The network structure potentially reflects the distinct pathophysiological cascades triggered by each virus.

We have adjusted the manuscript to explain better the importance of the analysis.

(See Lines 254-261; 266-268).

13. Why were you looking at the numbers of patients sharing two symptoms? Is this similar to a cluster or latent class analysis? Explanation might require supplemental material.

Its goal is to quantify symptom co-occurrence, which can then reveal syndromic patterns. It helps tell which symptoms tend to appear together, further hinting at possible underlying pathophysiological links; helps in hypothesis generation for using clinical signatures to distinguish the two infections. It also creates a visual network graph of how these symptoms relate in the patient population.

14. Specific comments: several symptoms require clarification and might be related to translation inaccuracy (lines 136-139): "adenomegaly" - should this be lymphadenopathy?; "conjunctival congestion" - conjunctival injection? (this is also duplicated so one should be deleted); "oropharyngeal changes" - what does this mean?

Thanks for this observation.

We wish to clarify that adenomegaly is also known as lymphadenopathy, and that both terms refer to the enlargement of one or more lymph nodes. The “oropharyngeal changes” in this case referred to oral mucosal lesions. Overall, we have since revised how the symptoms referenced in the comment have been listed. (See lines 120-123).

RESULTS

Reviewer #2:

15. Line 239 – What does it mean “by a consecutive sampling”? The fact that samples were collected at D1 and D28?

In this case, consecutive sampling referred to the methodology used for patient inclusion into the study. Here every individual who met the study inclusion criteria, signed the ICF during the recruitment period, and agreed to come for follow-up was selected for the study. There after their blood samples were collected at D1 and a follow-up collection at D28 post-inclusion. (Line 229-230).

16. Line 241 – Check the sentence: there is probably a duplication of verb.

The sentence has been reviewed and the repeated verb “were” deleted.

17. Line 243-248 – The paragraph is not very clear. Authors should make their results more readable. For example, there is the repetition of “on the admission,” as well as the symptoms associated with OROV infection are dispersed in across sentences, making difficult to understand which are important compared to DENV positive population.

Thanks for this observation. We have revised the paragraph to make it clearer. (See Lines 233-239).

18. Line 281-282 – Firstly, the verb “illustrated” is not proper for describing the outcome of clinically relevant assays: please change it. Most importantly, despite difference in ALT concentration between OROV and DENV patients, the mean (or median?) level is in the normality range: this is an important aspect to consider, because the benefit of such result is to support a possible virus-driven hepatic damage.

The normal ALT range is 7 - 56 U/L. However, it was noted that the ALT levels in OROV patients were significantly higher than among DENV patient. Although within normal ranges, the difference in ALT may be indicative of OROV’s hepatic involvement.

We have revised the paragraph to reflect this. (See Line 270-278).

19. Line 295-302 – Try to reduce data on sequencing quality to very essential ones; in addition, Figure 3 is not informative, with no added value on the results. Focusing on phylogenesis is recommended.

As advised, we have excluded the initial Fig 3 (Ct values and segments genome position). We summarized the sequencing results and focused instead on presenting the result on phylogenetic tree analysis (See Line 324-332; 335-338).

20. In figure 4 is not clear which the study sequences among.

We have improved on the resolution of this Figure (now Figure 3 in the revised manuscript).

21. Line 325-331 – Please rewrite the paragraph: the sentences are redundant, with the same scheme and words repeated. It should be more readable and harmonized.

We appreciate this observation, and have revised the genotyping results section, making it more clear and sensible (See Lines 324 -332)

22. Figure 5 is not informative.

The initial Fig 5 has been excluded in the revised manuscript.

23. Line 357-441 – There are a lot of data presented in this section. Even interesting, the presentation is not very clear. Please try to underline most important outcomes, to let readers to understand what are differences between DENV and OROV infections: such non-viral parameters could drive physicians in differential diagnosis.

We are thankful for the observations made, and agree with the reviewer. For clarity and ease of understanding, we have now condensed the section and opted to focus on the cytokine/chemokine profile between the two patient groups. (See Lines 341-365).

24. In addition: was there an equal distribution between the S1, S2, S3 groups to have reliable data on neurological markers? Even p-values supported data, a reduced cohort could limit the relevance.

We have since revised the section citing these groups. The distribution of headache severity is summarized in Table 1, where we note that patients with severe headache are twice as much in the OROV group than the DENV group.

We also acknowledge that a small sample size may affect the generalizability of the neurological markers involvement with headache intensity. However, the statistical significance in our initial analysis still holds, even after adjusting for sample size, suggesting robustness in the observed differences.

25. Figure 8 G-H is not very clear. Are they correlations between a non-viral parameter and headache intensity? How can be deduced the adjustment for age and gender?

These were correlations between leukocyte counts and the neuromarker α-synuclein pS129 against headache intensity, for each patient group. It is possible that age can significantly influence the overall correlation. Adjusting for gender may possibly water down the effect due to further subgrouping that may in turn diminish the sample size (n) analyzed.

Please note that in the new revised manuscript we opted to remove the Figure 8.

Reviewer #3:

26. The figures and tables should all be placed at the end of the manuscript and the titles and captions should be placed with the appropriate figures and tables. It's very hard to follow.

We have now included the tables in the text. As per the journal submission instructions, the figures were required to be uploaded separately.

27. Figures are very low resolution and are hard to read.

This we have rectified.

28. Lines 394-430 are impossible to understand - there is too much detail, terminology, and disconnected correlations. I don't know what any of it means.

In the revised manuscript, we have opted to remove the section on correlation and neuromarker analysis to maintain the work as clear and coherent as possible.

29. A single table or figure demonstrating the relevant correlations noted for OROV and DENV with the text highlighting the notable findings would be much more understandable.

Upon further consultation among ourselves and considering the general observations by the journal reviewers, we opted to exclude the correlation analysis and neuromarker results from the revised manuscript.

We are however thankful for the advice and will consider this approach in the next paper that will involve the neuromarker results and correlation with other clinical parameters.

30. There are places in the results where text should be in the methods or discussion - e.g., paragraph from lines 376-380: first sentence is methods; paragraph from lines 432-441: first sentence is methods; last sentence is discussion

Thank you for these observations. We have endeavored to improve on the overall quality of the manuscript. Please confirm with the revised version.

DISCUSSION AND CONCLUSION

Reviewer #2: Discussion

31. Some part of the discussion are a repetition of results: please change and adapt to a new form.

Thanks for your observation. We have revised the discussion and endeavored to improve the quality and clarity of the discussion.

32. Review the discussion according to changes in the above sections.

This has been done. Please confirm this in the revised version (See Lines 366 - 485).

Reviewer #3:

33. It's unclear whether the conclusions are supported by the data presented because the results are not presented in an understandable way as mentioned above.

The conclusion has been revised to include key findings from our study, as well as informed recommendations that may be explored in future research or by other investigators. (See Lines 482 - 509).

34. The sequencing data seem like a distinct topic from the rest of the paper. Can the authors connect this information to the clinical findings? Otherwise, I would recommend shortening the sequencing methods (or put in a supplemental section), results, and discussion.

We have tried to connect the sequencing/genome data with the current cohort of patients in the discussion section. We have related the current findings and observations with a previous study using a similar Manaus OROV isolate that reported increased infectivity and replication in this new lineage resulting from the reassortment event, compared to a much older historical strain (BeAn19991). (See Lines 373 - 390).

As for the clinical findings, it would have been difficult to do so considering that, we had no other OROV strain and patients with which to compare.

35. Would not present new data in the discussion (line 488).

This has been rectified.

36. There is little discussion about the differences in symptoms (other than headache) between Oropouche virus disease and dengue.

We have revised the discussions on the symptoms opting to focus on the symptoms with a significant difference between the two diseases (See Lines 392 - 411).

37. Overall, the discussion needs to be streamlined and organized by topic as discussed below.

Thanks for this observation. We have endeavored to streamline the discussion on basis of the key findings. We hope that the revised version is to the reviewer’s liking.
